# Evaluation of Two-Assay Serological Testing Strategies for Anti-HCV Screening in Italian Populations: A Dual Screening Approach

**DOI:** 10.3390/diagnostics14060570

**Published:** 2024-03-07

**Authors:** Elena Zocca, Silva Seraceni, Teresa Cafaro, Tamara Emanuela Cervone, Laura Cardarelli, Massimiliano Valisi, Isabella Polidori, Massimo Pieri, Flaminia Tomassetti, Francesco Broccolo

**Affiliations:** 1 Cerba HealthCare Italia Rete Diagnostica Italiana, 35020 Limena, PD, Italy; 2 Cerba HealthCare Italia, Via Durini, 20139 Milan, MI, Italy; 3 Cerba HealthCare Italia Srl, 00012 Guidonia, RM, Italy; isabella.polidori@cerbahealthcare.it (I.P.);; 4 Department of Laboratory Medicine, “Tor Vergata” University Hospital, 00133 Rome, RM, Italy; 5 Department of Experimental Medicine, University of Salento, 73100 Lecce, LE, Italy

**Keywords:** HCV, hepatitis C virus, antibody, anti-HCV antibody, hepatitis, laboratory algorithm, two-assays strategy

## Abstract

(1) Background: Hepatitis C virus (HCV) screening mostly uses a one-assay anti-HCV testing approach, which has a higher probability of false-positive results in populations with low HCV prevalence. (2) Methods: In this investigation, 17,926 participants were screened for HCV, and the reactives were tested using a two-assay anti-HCV approach: Elecsys ElectroChemiLuminescence (ECL) and a ChemiLuminescence ImmunoAssay (CLIA), respectively. A recombinant immunoblot assay (RIBA) was performed to confirm anti-HCV positivity. Statistical analysis was performed. (3) Results: A total of 350 specimens were reactive in the ECL screening, of which CLIA retesting showed that 292 (83.4%) were anti-HCV reactive (283 positives, 9 indeterminate, none negative by RIBA), but 58 (16.6%) were not anti-HCV reactive (15 positive, 12 indeterminate, 31 negatives by RIBA). The two-assay strategy significantly improved the positive predictive value (PPV: 95.00%) with χ^2^: 7.59 (*p* < 0.01) compared to the PPV assessed by one assay (PPV: 90.6%) with χ^2^: 34.51 (*p* < 0.001). The ROC curve defined a sensibility and specificity for the dual approach of 99.66% and 100.00%. (4) Conclusions: Compared with a one-assay testing strategy, the two-assay testing strategy may significantly reduce false positives in anti-HCV testing and identify inactive HCV infection in low seroprevalence populations.

## 1. Introduction

Hepatitis C virus (HCV) infection is one of the leading causes of liver cirrhosis, hepatocellular carcinoma, and mortality worldwide [1,2,3]. Most of the people affected by hepatitis C do not exhibit any symptoms; many of them are unaware that they are infected with hepatitis C until liver damage emerges, years or even decades after the virus infection [4,5]. The infection can manifest in both acute and chronic phases (85% of cases). The acute phase can present widely differing symptoms due to the interaction of the immune system with the virus and the antigen exposure. About 15% to 25% of individuals with an acute HCV infection typically undergo spontaneous clearance within 6 months, marked by undetectable serum HCV RNA and normalization of ALT levels. Conversely, approximately 75% to 85% of these individuals progress to chronic HCV infection, distinguished by the persistence of HCV RNA in the blood for more than 6 months following the onset of the acute infection [6]. Lastly, it can lead to fulminant hepatic failure; however, in most cases, it results in undiagnosed infections initially, characterized by subclinical and flu-like symptoms, but diagnosed only in late stages [7]. The delayed diagnosis leads to the chronicization of this pathology, which tends to re-emerge (usually after the age of 50), progressing to hepatic cirrhosis (80%) and, in the worst cases, hepatocellular carcinomas [8].

Globally, 71 million people have a chronic HCV infection, and 1.75 million new infections occur each year. However, in low- and middle-income countries and high-income settings, respectively, only 20% and <1% of patients received a diagnosis and treatment [9].

Hepatitis C prevalence is now estimated to be 2.8% worldwide, with significant regional and demographic variations [10]. Egypt is thought to have the greatest global prevalence of HCV, at about 12%, whereas Iran has the lowest prevalence, at 0.30% [10]. In the Italian population, HCV prevalence increased in generations born before the 1950s due to the post-war HCV epidemic. However, over the last forty years, improvements in hygienic conditions, including using disposable sanitary materials, blood transfusion control, and population awareness through anti-HCV screening surveillance programs and educational initiatives, have led to a significant decrease in HCV rates. Furthermore, since 2019, Italy has adhered to the hepatitis C virus (HCV) elimination project by 2030, following the WHO recommendation [10,11], even if approximately 2 million subjects are still tested reactive for the HCV, i.e., carriers of anti-HCV antibodies, of which 250 thousand subjects with chronic hepatitis are under antiviral treatment [12,13].

The majority of prior research has been on HCV screening methods for groups who are at high risk, such as alcoholics, drug users, men who have sex with other males [14], and HIV-positive patients or hepatitis B virus (HBV), whereas very few studies have focused on screening methods for broader populations. Several studies have shown that chronic alcohol use is one of the most important external risk factors for the progression of chronic hepatitis C to cirrhosis and hepatocellular carcinoma (HCC) [15,16,17]. Chronic HCV infection can lead to from 10 up to 20 years of liver cirrhosis in 10–20% of individuals [18].

The World Health Organization (WHO) has created evidence-based recommendations that concentrate on who should be tested for chronic hepatitis C infection and how to conduct the test [19], i.e., the serological assay approach and the right period to be tested. 

Firstly, the diagnosis and monitoring of HCV infection are based on two types of tests: a serological test that detects HCV antigen-specific antibodies, and tests that detect viral RNA or HCV core antigens [20]. Then, it is crucial to clarify that the detection of the anti-HCV antibodies through serum analysis should be conducted within a specific timeframe. Performing the antibody detection test beyond six months after the presumed infection may yield a false-negative result [21]. However, using just one serological test makes no distinction between active infections and resolved ones; therefore, it is necessary to confirm the data with Western blot analysis, still the gold standard for serological detection of HCV. In addition, false-negative results are frequent due to a long window period, the period from initial infection to seroconversion, which lasts between 45 and 68 days. False-positive results from serological tests may occur due to interfering factors, including high gamma globulin levels, nephritic syndrome, pregnancy, autoimmune diseases, or viral or parasitic infections [22]. Through rapid diagnostic tests (RDTs) or laboratory-based serum samples, such as enzyme immunoassays (EIAs), electrochemiluminescence immunoassays (ECLs), and chemiluminescence immunoassays (CLIAs), the current diagnostic algorithm prioritizes the detection of HCV antibodies as evidence of past or current HCV infection. To determine whether there is an HCV infection with viremia, further testing for HCV RNA or HCV core antigen is necessary. In order to reduce the need for additional tests (confirmatory test, such Western blot) and reduce the likelihood of reporting false-positive findings, the ideal S/CO ratio in a variety of laboratory-based serological assays has been studied in the past [23,24]; however, the results were not consistent [24,25]. In light of these reasons, our hypothesis was to use a dual approach using two serological screening assays, and also to minimize the turnaround time (TAT) and laboratory costs. The rate of HCV eradication could only be significantly accelerated by widespread population screening.

The low diagnosis rate of this infection is mainly due to the lack of national policies and/or guidelines for the diagnosis of the infection, expensive and complex diagnostic assays, and poor acknowledgment in the population and persisting prejudice for these types of infections.

The early identification of individuals with chronic HCV infection is crucial and enables rapid initiation of necessary treatments, which could help prevent or delay the progression of liver diseases. Early intervention increases the probability of achieving a sustained viral response (SVR), indicating successful treatment in eliminating the virus from the body. Likewise, this contributes to reducing the mortality rate associated with HCV-related liver pathologies. Therefore, since an early and prompt diagnosis of HCV infection is needed for the prevention, care, and treatment of the patients, the role of the laboratory is extremely important. For these reasons, our study aimed to validate and assess a laboratory algorithm for the screening of HCV antibodies. In particular, the purpose is to investigate the reliability of two commercially available anti-HCV antibody kits used in routine laboratory testing in Italy: the electrochemiluminescence immunoassay (ECL) (Cobas e 801^®^ Elecsys Anti-HCVII, Roche) and the chemiluminescence immunoassay (CLIA) (Liaison XL^®^ Murex HCV Ab, Diasorin), in comparison with the Recombinant Immunoblot Assay (RIBA), nowadays the gold standard for the HCV screening. The approach with the two assays should avoid the confirmation test with the Western blot, reducing time and cost and limiting the operator-dependent work, using just automatized systems.

## 2. Materials and Methods

### 2.1. Study Design

A total of 17,926 serum samples were collected from subjects who adhered to anti-HCV screening from January 2021 to July 2022. Among them, 6453 (36%) were males with a mean age of 54.5 ± 18 years, and 11,473 (64%) were females with a mean age of 44 ± 16 years. All serum samples were analyzed for anti-HCV screening at Cerba Healthcare Italia in Italy. 

The first anti-HCV testing was using electrochemiluminescence (ECL) immunoassay systems within four hours after collection.

Each anti-HCV-reactive serum sample was divided into 2 aliquots (each 500 μL) to retest them using the second serological assay (MUREX HCV Ab), chemiluminescence (CLIA), and at the end the recombinant immunoblot assay (RIBA) to confirm the results obtained, following the national guidelines (ISTISAN 06/47).

The order of the two methods was selected by the specificity of the two tests, as declared by the manufacturer, first analyzing the assay with higher specificity (ECL: 99.96% ref. for European population) in order to cut out all the negative samples, and secondly the assay with lower specificity (CLIA DiaSorin: 99.5%).

To minimize variations related to time or storage, the different tests were performed on the same samples’ aliquot for 1–2 h maximum from the first test. The other was stored at −80 °C for RIBA, which was performed as a supplemental test to confirm anti-HCV positivity within 7 days, following the stability ranges defined by the recommendations of the manufacturers.

The negative cases by CLIA assay and the indeterminate cases by RIBA were also tested with a nucleic acid test (NAT) to determine the results in the discrepancies.

All experimental procedures were conducted according to the Declaration of Helsinki.

### 2.2. ECL Immunoassay for Anti-HCV

An ECL immunoassay was applied for anti-HCV testing using the Elecsys anti-HCV II assay on the Cobas 801 analyzer (Roche Diagnostics, Mannheim, Germany). The kit was a third-generation test using peptides and recombinant antigens representing the core, NS3, and NS4 to capture the corresponding antibodies.

The results were expressed as signal-to-cutoff (S/CO) ratios: S/CO < 1.0 indicated anti-HCV nonreactivity, and S/CO ≥ 1.0 indicated anti-HCV reactivity. All S/CO ≥ 1.0 sera were retested in duplicate according to the manufacturer’s instructions. If either of the two results remained S/CO ≥ 1.0, then the subject was considered anti-HCV reactive. The anti-HCV reactive sera were retested using a CLIA anti-HCV reagent kit. The sensitivity and the specificity were, respectively, 100% and 99.96% in a European cohort, as declared by the manufacturer. Quality control was performed before any analytical investigation.

### 2.3. CLIA Immunoassay for Anti-HCV

An indirect CLIA immunoassay was applied for anti-HCV testing using LIAISON XL Murex HCV Ab (DiaSorin SpA, Saluggia, Italy), based on two recombinant antigens (core and NS4) specific for HCV that are used for coating magnetic particles (solid phase), and a third ready-to-use aqueous HCV antigen (biotinylated NS3). The samples were analyzed on Liaison XL (DiaSorin SpA, Saluggia, Italy).

The results were expressed as signal-to-cutoff (S/CO) ratios: S/CO ≥ 1.0 indicated anti-HCV reactivity, samples with S/CO values ≥ 0.80 and <1.00 were considered equivocal, and S/CO < 1.0 anti-HCV nonreactivity, according to the manufacturer’s instructions. If either of the two results remained S/CO ≥ 1.0, then the subject was considered anti-HCV reactive. The sensitivity and the specificity were, respectively, 100% and 99.5% in the hospitalized population, as declared by the manufacturer. Quality control was performed before any analytical investigation.

### 2.4. Recombinant Immunoblot Assay for Anti-HCV

Specimens with reactive anti-HCV results were further tested with RIBAs to confirm anti-HCV positivity using a recombinant immunoblot kit for antibodies against the hepatitis C virus (INNO-LIA HCV Score, Beijing Wantai Biopharm, Beijing, China). The nitrocellulose strips contained seven bands for the core, NS3, NS4-1, NS4-2, and NS5 antigens as well as control A and control B. The result was defined as negative, ±, 1+, or 2+ by comparing the color of the antigen band with control A. Anti-HCV positivity was defined as the presence of at least two antigens with greater than or equal to 1+ (≥1+) reactivity. An indeterminate result was defined by only one band scoring ≥1+. Anti-HCV negativity was defined by the absence of antigens scoring ≥1+. The tests were performed according to the manufacturer’s instructions by expert technicians.

### 2.5. Statistical Analysis

Statistical analysis was performed with MedCalc Software Ver.18.2.18 (MedCalc Software Ltd., Ostend, Belgium).

The data are expressed in positive predictive value (PPV) and negative predictive value (NPV). The statistical significance level established for all tests performed gave a *p*-value of (*p*) < 0.05. The frequency data were analyzed using the two-way chi-square (χ^2^) test analyzing the relationship between two classification factors (reactive vs. non-reactive for both the assay). The χ^2^ test was calculated and, where possible, a Cochran–Armitage was applied to test for trend.

The receiver operating characteristic (ROC) curves were calculated for the two assays, categorized by RIBA results, from which the reactivity cut-offs were extrapolated. The ROC curve is an analytical method that represents graphically the performance of a binary diagnostic classification method, interpreting the data from a dichotomous (0–1) form to assess the presence or the absence of the sepsis [26].

For each ROC curve generated (reported as the area under the curve, AUC), a confidence interval (CI) was assumed by 95%. The cut-off was determined by calculating the Youden Index, a summary measure of the effectiveness of the ROC curve.

The Shapiro–Wilk test was used to verify the data distribution as it is applied to a sample wherein the null hypothesis is that the sample has been generated from a normal distribution. If the data showed a non-Gaussian trend, the Mann–Whitney test was used, being the non-parametric alternative test to the independent sample *t*-test. The differences of the data collected between the various groups were represented using median and 1st and 3rd interquartile (InterQuartile Range, IQR).

## 3. Results

### 3.1. One-Assay Serological Testing Strategy Using ECL Screening

**Figure 1:**
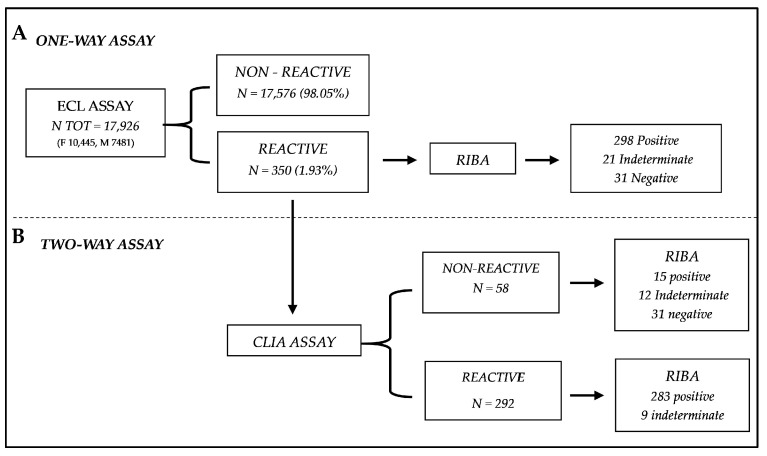
Diagram explaining the algorithm utilized. Of the total analyzed samples (*n* = 17,926) in the chosen period, the samples (*n* = 350) reactive to the ECL assay were also screened with another screening method, the CLIA assay. (**A**) From total samples, 350 reactive samples were assessed by the ECL assay, and 298 positive, 31 negative, and 21 indeterminate were confirmed with the RIBA analysis. (**B**) The 350 reactive samples were also tested with CLIA assay, resulting on 292 reactive samples and 58 non-reactive. From 292 samples, just 283 positive results were confirmed by the RIBA, and the remaining 9 were evaluated as indeterminate. [N TOT: Total number; M: males; F: females; ECL: Electrochemiluminescence Immunoassay; CLIA: Chemiluminescence Immunoassay; RIBA: recombinant immunoblot assay].

A total of 17,926 subjects were screened with the first serological method, with a mean age of 53 ± 16 (min. 18, max. 90) years old, and 58% (10,445 out of 17,926) were women and 42% (7481 out of 17,926) were men. Among the 17,926 subjects, just 1.93% (350 out of 17,926) showed anti-HCV reactivity in the ECL assay, of which no cases failed to show reactivity in a subsequent repeated test with the same assay. Therefore, a total of 350 cases were included in this study, and the results are reported in Figure 1. Among these sera, RIBA showed that 298 were confirmed as anti-HCV positive, 21 were indeterminate, and 31 were negative. According to the manufacturer’s instructions, samples with S/CO ≥ 1.0 were considered positive for anti-HCV, with a PPV of 90.60%, as reported in Table 1.

### 3.2. Two-Assay Serological Testing Strategy Using CLIA Testing, after ECL Screening

The 350 specimens that were anti-HCV reactive on ECL were retested using the second serological assay (CLIA), as reported in Figure 1. The second method used showed that 83.4% (292 out of 350) were anti-HCV reactive, while the other 16.6% (58 out of 350) specimens were non-reactive. Among 292 specimens that were anti-HCV reactive on both serological tests, RIBA showed that 283 were anti-HCV positive, 9 were indeterminate, and none were negative, for a PPV of 95.00% (Table 1), higher than the PPV of the one-assay serological test. Among the 58 sera (non-reactive for CLIA) with inconsistent results, 15 were anti-HCV positive, 12 were indeterminate, and 31 were negative by RIBA, for an NPV of 77.5% (Table 1).

The χ^2^ test was performed to evaluate how likely the frequencies of positive and negative rates for one-assay would be presented in the population (the RIBA results). The χ^2^ test evaluated was 34.51 (*p* < 0.001). Then, the χ^2^ test was performed again to evaluate how likely the frequencies of positive and negative rates for two-assay would be presented. The second χ^2^ test evaluated was 7.59 (*p* < 0.01). The χ^2^ results are reported in Table 1.

The indeterminate results by RIBA were not included in these analyses.

### 3.3. ROC Analyzing ECL Screening and CLIA Retesting

**Figure 2:**
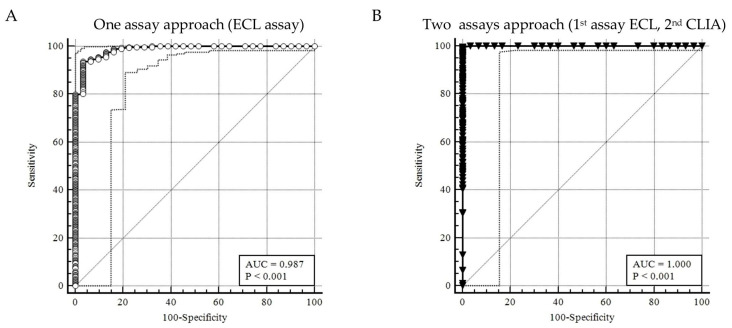
(**A**) ROC curve performed on the results obtained from the one assay: ECL assay. (**B**) ROC curve performed on the results obtained from the two-assay: ECL assay as 1st method and CLIA assay as 2nd method. [ECL: Electrochemiluminescence assay; CLIA: Chemiluminescence assay; AUC: Area Under the Curve].

Figure 2 illustrates the ROC curves of the ECL assay (A) and the CLIA assay (B). The ROC curve for the first method showed a sensitivity of 93.62% and a specificity of 96.77% with an AUC of 0.987 (*p* < 0.001). Instead, the second method achieved a sensitivity of 99.66% and a specificity of 100.00% with an AUC of 1 (*p* < 0.001). The Youden index was calculated for the ECL assay as 0.90 with a cut-off for reactivity to the test of >15.70; meanwhile, the Youden index was calculated for the CLIA assay as 0.99 with a cut-off for reactivity of >0.33.

**Figure 3:**
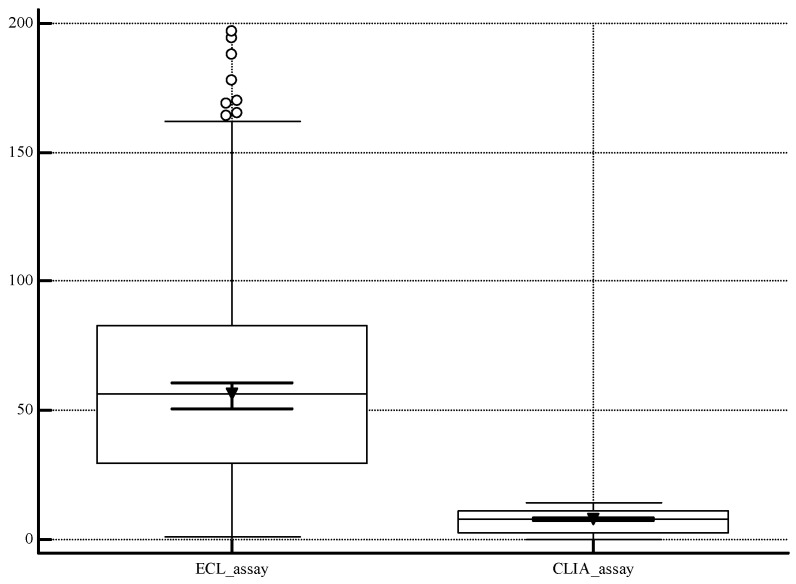
Box plot graph representing the data distribution of the results of the two assays. [ECL: Electrochemiluminescence assay; CLIA: Chemiluminescence assay].

Figure 3 highlighted the difference between the data from the two assays: median 56.70 S/CO [Interquartile Range, IQR: 29.50–83.04] for one-assay analysis; median 7.80 S/CO [IQR: 2.90–11.00] for the two assays analysis.

## 4. Discussion

The global initiative to eradicate the hepatitis C virus (HCV) infection has led to the implementation of screening strategies aimed at identifying and managing infected individuals. In our study, we adopted a comprehensive approach involving an ECL screening for HCV, followed by a second screening method, a CLIA assay, aligning with similar strategies employed for other viruses; actually, in recent years the prevention and diagnosis of HIV infection has been used by non-profit organizations, such as ANLAIDS in Italy [27,28]. In the event of a positive result, the test is repeated using a first-level screening test, and if confirmed, further testing for HIV RNA is conducted [29].

The diagnostics and clinical laboratories play an essential role in the identification of HCV reactive cases and in the reduction of the HCV infection rate and hepatitis-related mortality [30]. In this global context for the eradication of HCV infection, a cohort of 17,926 patients underwent testing from January 2021 to July 2022 using ECL screening for HCV. Of these, 350 patients tested positive and were subsequently retested using a second screening test (CLIA assay).

The study’s findings confirmed the appropriateness of the dual testing approach: the use of just one screening method achieved a sensibility and specificity of 93, 62% and 96.77%, results consistent with other precedent studies [31,32]; meanwhile, the use of two screening methods improved the sensitivity to 99.66% and the specificity to 100%, as shown in Figure 2. This high level of accuracy underscores the effectiveness of utilizing two different screening methods to ensure reliable results. Despite the overall success of the dual testing approach, a slight overestimation was observed in the screening methods, particularly in +3.18% (292 out of 283) of cases that the RIBA detected as negatives. Nevertheless, the percentages of false positives in Table 1 after the two-method testing was significantly decreased (PPV: one assay 90.60 vs. two assay 95.00), suggesting the excellent performance of the strategy. In addition to the PPV, the χ^2^ analysis in Table 1 proved that the dual approach could better manage the discrimination of positive and negative results in the population studied (χ^2^ test: one assay 34.51, *p* < 0.001 vs. two assay 7.59, *p* < 0.01).

These last results overturn the data previously evaluated in another work [33], which showed no significant ability to discriminate false positive rate between one assay and two assays. It is probably that the discordant findings are related to the different methods used—our study utilized CLIA instead of Chemiluminescence Microparticle Immunoassay (CMIA)—and the different testing order. Our approach was to use a more stringent second test with higher sensitivity and specificity, declared by the manufacturers to give a faster but accurate response, and minimize the laboratory’s costs, due to the decrease in RIBA evaluation for all the positive samples to the screening test. 

Although, the world has focused its attention mostly on the detection and treatment of HCV infection [10], less effort and investment has been made to ensure accurate and affordable diagnostic tools. Ironically, in many settings, the prohibitive costs of HCV diagnostics often exceed the cost of curative therapy [34]. The dual approach investigated in our study could minimize the cost and the TAT, improving the laboratory routine for rapid, easy, and economical HCV diagnostics. The use of two assays could reduce the need for a confirmatory test like the RIBA that, despite still being the method of choice for serological assays due to the higher PPV, has a higher cost and increased likelihood of indeterminate results that underscore some limitations. Specifically, the combined reactivity results for both of these two tests proved to have a clear ability to identify all the positive samples. On the other hand, a notable diagnostic limitation is observed in the discordant cases, i.e., the samples that resulted are reactive for ECL assay but non-reactive for CLIA assay (*N* = 58, Figure 1). In these discordant cases, several outcomes were observed after the RIBA execution: 15 positive, 21 indeterminate, and 31 negative. The discordant cases will be reported with the result obtained by RIBA analysis. This diversity of results limits the full feasibility of the diagnostic approach hypothesized; as for all those samples with different results between the two assays, a second-level analysis with RIBA or a direct molecular NAT analysis must still be carried out. Anyhow, the initial hypothesis of employing two primary-level tests for analyzing serological samples for HCV is still satisfied, aiming to minimize the use of RIBA in the clinical laboratory. Cases resulting in reactivity for both methods conclusively affirm the positivity of the respective samples. This certainty supports the reduction or potential substitution of RIBA in the diagnostic laboratory workflow. By adopting this approach and carrying out the RIBA only for the few discordant cases (58 out of 350), laboratory costs and times could be significantly lowered. This optimized approach allows for the streamlining of HCV screening while saving the capability to identify all infected individuals. The data observed highlighted the effectiveness of the combined tests in confirming true positives (PPV: 95%). Even still, it is necessary to remember that the discordant results underlined the necessity to continue to use the confirmatory tests. Continuous investigation and validation of the diagnostic pathway are essential for ensuring the reliability and accuracy of HCV screening protocols.

Moreover, it is important to recognize the limitations associated with the screening tests utilized in the study, e.g., variables such as cross-reactivity, time testing, or different antigens detected, that could influence some results. In Figure 3, we would represent the huge difference between the medians of the results for the two methods (ECL: 56.70 S/CO; CLIA: 7.80 S/CO), due to the detection of distinct antigens (ECL: core, NS3, and NS4; CLIA: core and NS4), which could cause potential challenges in reporting. Consequently, the application of this dual testing strategy in routine laboratories may lead to the release of results only in qualitative information. Another limit is the lack of information about the anamnesis for HCV-reactive patients and a stratification of the population.

## 5. Conclusions

It is important to remember that the prevention of hepatitis C depends exclusively on reducing the risk of exposure to the virus in healthcare facilities and populations at highest risk through sensibilization campaign and screening diagnostics tests. The lack of an HCV vaccine capable of generating cross-neutralizing antibodies directed against the epitopes of HCV genotypes and inducing cell-mediated immune responses is still, unfortunately, not available. Therefore, an active screening of the population, through serological tests and some laboratory approaches to minimize the cost and the time, could nowadays be an important aim in the fight against hepatitis C. Further, research should explore the adaptability of this dual testing strategy across diverse populations and settings. Additionally, assessing the economic implications, feasibility, and scalability of this approach will be crucial for its integration into routine healthcare practices. Continued collaboration between researchers, healthcare providers, and policymakers is essential to refine and implement strategies that contribute to the global goal of eradicating HCV.

## Figures and Tables

**Table 1:** Positive and negative predictive value and χ^2^ test using one vs. two serological assays [RIBA: Recombinant Immuno Blot Assay; PPV: Positive Predictive Value; NPV: Negative Predictive Value].

Classification	Confirmed Positive by RIBA	Confirmed Negative by RIBA	Indeterminate for RIBA	PPV	NPV	χ^2^ Test	*p*-Value
*One assay*	298	31	21	90.6%	ND	34.51	<0.001
*Two assays*	283	31	12	95.00%	77.50%	7.59	<0.01

## Data Availability

The data presented in this study can be obtained by contacting the corresponding author with a reasonable request.
